# Mechanical Signaling in the Pathophysiology of Critical Illness Myopathy

**DOI:** 10.3389/fphys.2016.00023

**Published:** 2016-02-04

**Authors:** Rebeca C. Kalamgi, Lars Larsson

**Affiliations:** ^1^Basic and Clinical Muscle Biology, Department of Physiology and Pharmacology, Karolinska InstitutetStockholm, Sweden; ^2^Department of Clinical Neuroscience, Clinical Neurophysiology, Karolinska InstitutetStockholm, Sweden

**Keywords:** critical illness myopathy, mechanical silencing, mechanotransduction, sarcomere, mitochondria, mTORC1

## Abstract

The complete loss of mechanical stimuli of skeletal muscles, i.e., the loss of external strain, related to weight bearing, and internal strain, related to the contraction of muscle cells, is uniquely observed in pharmacologically paralyzed or deeply sedated mechanically ventilated intensive care unit (ICU) patients. The preferential loss of myosin and myosin associated proteins in limb and trunk muscles is a significant characteristic of critical illness myopathy (CIM) which separates CIM from other types of acquired muscle weaknesses in ICU patients. Mechanical silencing is an important factor triggering CIM. Microgravity or ground based microgravity models form the basis of research on the effect of muscle unloading-reloading, but the mechanisms and effects may differ from the ICU conditions. In order to understand how mechanical tension regulates muscle mass, it is critical to know how muscles sense mechanical information and convert stimulus to intracellular biochemical actions and changes in gene expression, a process called cellular mechanotransduction. In adult skeletal muscles and muscle fibers, this process may differ, the same stimulus can cause divergent response and the same fiber type may undergo opposite changes in different muscles. Skeletal muscle contains multiple types of mechano-sensors and numerous structures that can be affected differently and hence respond differently in distinct muscles.

## Introduction

The complete loss of mechanical stimuli in skeletal muscles, i.e., the loss of external strain, related to weight bearing, and internal strain, related to the contraction of muscle cells, is uniquely observed in pharmacologically paralyzed or deeply sedated mechanically ventilated intensive care unit (ICU) patients. This mechanical silencing is an important factor triggering the specific critical illness myopathy (CIM; Ochala et al., 2011a; Llano-Diez et al., 2012; Renaud et al., 2013). Acquired muscle weaknesses in the ICU is a major complication that occurs in severely ill patients and has significant impact on the immune system, amino acid reserves, and temperature regulation (Puthucheary et al., 2010). Muscle wasting and weakness in the ICU is increasingly being recognized and the increasing prevalence of diagnosed ICU acquired weakness is both due to a growing awareness and improved survival of patients with prolonged organ failure (Puthucheary et al., 2010). For these reasons, a thorough understanding of the molecular mechanisms that regulate skeletal muscle mass is important for development of effective rehabilitation programs and possible pharmacological interventions that can prevent or alleviate the loss of skeletal muscle mass and function (Isaacson and Brotto, 2014).

CIM affects limb and trunk skeletal muscles resulting in quadriplegia due to a preferential myosin loss, muscle atrophy, reduced muscle membrane excitability, and unbalanced muscle metabolism (MacFarlane and Rosenthal, 1977; Rich et al., 1997, 1998a,b; Larsson et al., 2000; Sander et al., 2002). As a consequence, the illness often complicates weaning from the ventilator and increases the length of the stay in the ICU. This results in prolonged immobility, need for intensive rehabilitation and increased risk for pneumonia, morbidity, mortality, and negative economic consequences for health careproviders. Muscle atrophy occurs within 10–21 days of muscle disuse when healthy older adults are bound to bedrest. Similar adverse skeletal muscle changes happen as early as day 5 to 7 after ICU admission in critically ill patients (Puthucheary et al., 2010).

Many triggering factors have been suggested for CIM and muscle wasting in the ICU including sepsis, systemic inflammatory response syndrome (SIRS) and multiple organ failure. Animal models have, however, allowed the separation of sepsis and other confounding risk factors, e.g., mechanical ventilation and muscle unloading, which are usually present in ICU patients (Banduseela et al., 2009; Ochala et al., 2011b; Aare et al., 2013). Sepsis on its own has not been able to replicate the CIM phenotype (Friedrich et al., 2015). Other potential triggering factors evolve from the interventions used in modern anesthesiology and in the ICU, that is; prolonged mechanical ventilation, neuromuscular blockers (NMB), systemic corticosteroid hormone treatment, and muscle unloading (Larsson, 2007).

Although the muscle impairment associated with CIM is being described with increasing accuracy as a result of advances in genetic and proteomic science, the exact cause and underlying mechanisms of the disease remain unknown. The loss of skeletal muscle mass is a complex process that occurs as a consequence of a variety of stressors. Atrophy includes the reduction of muscle fiber cross sectional area (CSA) due to a net loss of proteins, organelles and cytoplasm. This occurs as a result of alterations in the balance between anabolic and catabolic processes, with the net result being a loss of muscle mass when protein breakdown exceeds protein synthesis (Bodine and Baehr, 2014). Acute muscle atrophy occurs in many pathological conditions, including neural inactivity, mechanical unloading, inflammation, metabolic stress, and elevated glucocorticoids levels. Acute muscle atrophy is then due to hyper-activation of the main cellular degradation pathways like the ubiquitin-proteasome system (UPS) and autophagy lysosome pathways (Sandri, 2013; Schiaffino et al., 2013). However, it is also well established that muscle unloading induces a rapid decrease in protein synthesis which contributes to the loss of muscle mass (Thomason and Booth, 1990; Han et al., 2007).

## Muscle unloading and cellular mechanotransduction

**Figure 1:**
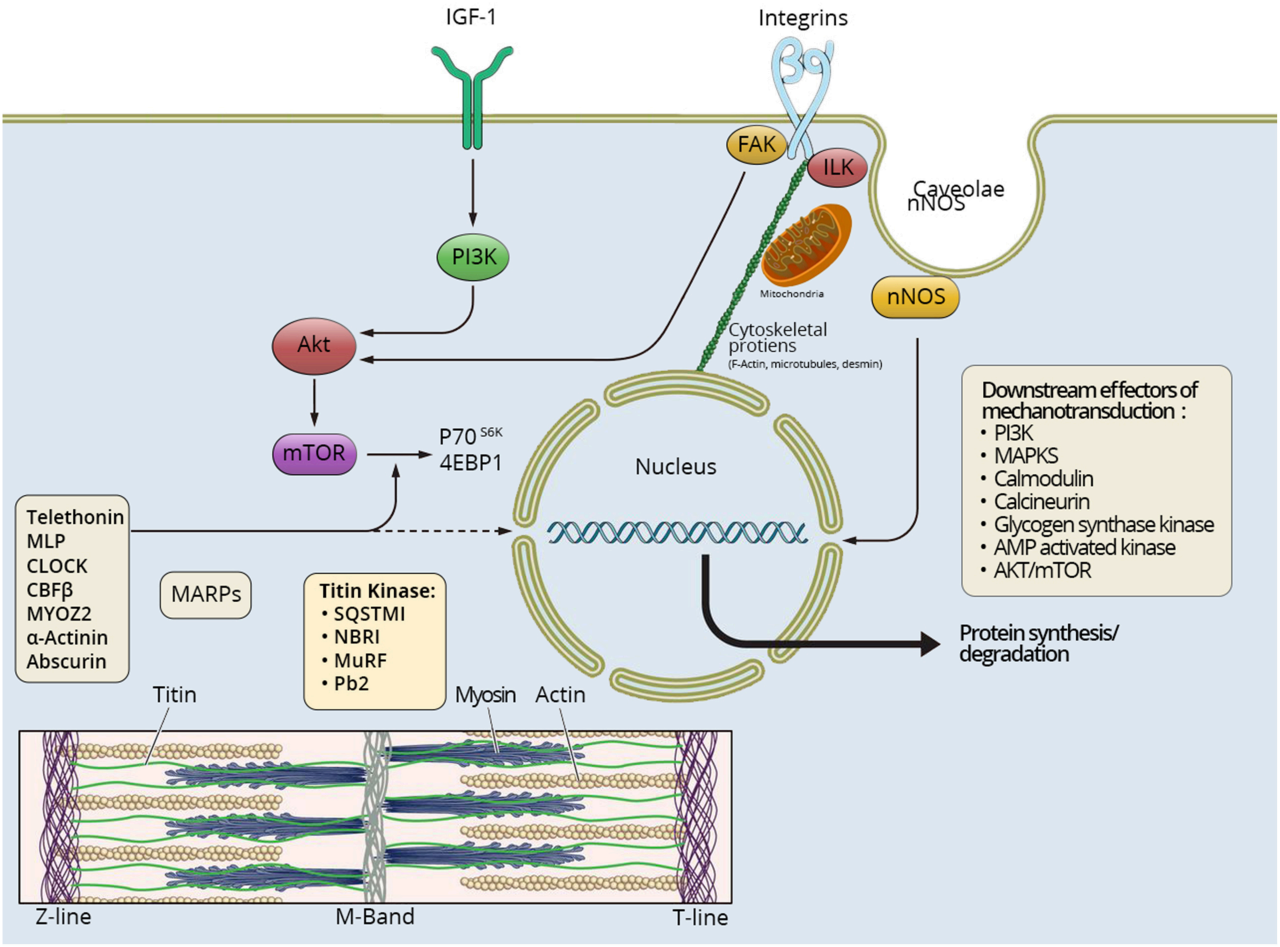
**Mechanosensing in skeletal muscle**. Different pathways involved in mechanosensing and tensegrity in skeletal muscle are briefly summarized. Multiple signaling pathways influence protein synthesis and degradation in the muscle fiber spanning from the muscle membrane and extracellular matrix to the M-band in the center of the sarcomere. IGF-1 has been suggested to play an important role for the muscle hypertrophy induced by mechanical overload. In addition caveolae, respond to cell stress and stretch-induced signaling, and many different proteins involved in cell signaling bind to caveolins, such as neural nitric oxide synthase (nNOS), tyrosine kinases, small GTPases, and growth receptors. Integrins spanning from the extacellular matrix to the interior of the muscle cell, linked to cytoskeletal actin, directly connect to the nuclei and mitochondria, thus allowing a “hard-wired” and rapid signal propagation to nuclear and mitochondrial DNA. A number of sarcomeric proteins are involved in mechanosensing, and there is emerging evidence of a very dynamic exchange of multiple sarcomeric proteins to the cytoplasmic pool affecting muscle gene expression in response to mechanical load from the Z-line to the center of the sarcomere in the M-band. Multiple major signaling cascades are downstream effectors of mechanosensing, such as PI-3K, MAPKs, calmodulin, calcineurin, glycogen synthase kinase, AMP activated kinase, and Akt/mTOR.

In order to understand how mechanical tension regulates muscle mass, it is critical to know how muscles sense mechanical information and convert stimulus to intracellular biochemical actions and changes in gene expression, a process called cellular mechanotransduction (Goldberg, 1968; Goldberg et al., 1975; Vandenburgh, 1987; Ingber et al., 2014). Skeletal muscle contains multiple types of mechano-sensors with diverse responses to changes in tension (Gautel, 2011a). Structures involved in mechanotransduction include integrins, sarcolemmal structure proteins, caveolae, cytoskeletal proteins, mitochondria, and sarcomeric proteins spanning from the Z-disc in to the M-line in the center of the sarcomere (Figure 1).

### Mechanotransduction pathways

Integrins are a large family of cell surface receptors that links the extra cellular matrix (ECM) to the cytoskeleton, regulating many cellular functions including cell proliferation, migration, and differentiation (Ingber, 1991; Giancotti and Ruoslahti, 1999). Integrins work both as mechanotransducers and adhesive receptors that can signal trough the cell membrane in both directions. ECM proteins can bind to integrin receptors and then form a cluster at the level of the cell membrane. The cytoplasmic tail of integrins can further associate with various intracellular proteins which then physically link the integrins to the internal cytoskeleton. The anchorage complex of ECM proteins, integrins, and cytoskeletal proteins is known as focal adhesion that ranges from one side of the cell membrane to the other (Wary et al., 1996; Giancotti and Ruoslahti, 1999; Wei et al., 1999; Graham et al., 2015). By forming focal adhesions, integrins allow mechanical forces to be transmitted to membrane proteins. Integrins also interact with mechano sensitive ion channels in focal adhesions (Martinac, 2014).

Caveolae are 60–80 nm cup-shaped invaginations of plasma membrane formed by caveolins. They are present in many cells but are particularly abundant in muscle cells, adipocytes, and endothelial cells. Sinha and colleges established that cells respond to an acute mechanical stress through rapid flattening of caveolae into the plasma membrane (Sinha et al., 2011). Stress-induced flattening of caveolae is strictly passive whereas the formation of invaginated caveolae is energetically favored through interactions with cortical actin in the presence of ATP. The caveolae invaginations are supported by extracellular matrix (ECM), which serves as external support, and the cytoskeleton, whose contraction and expansion locally regulate the mechanosensitivity of mechanosensitive channels in animal and human cells.

Wang and co-workers, were able to demonstrate that mitochondria also play an essential role in mechanotransduction. By confocal microscopy they confirmed that mitochondria associate directly with microtubules and distribute throughout the entire cytoplasm (Wang et al., 2001). In addition to microtubules, other cytoskeletal proteins, such as the intermediate filament desmin, are connected to mitochondria to provide a coordinated movement and alignment throughout the depth of the cell (Rappaport et al., 1998; Milner et al., 2000). When a mechanical pulling stress is applied to integrins, mitochondria show movement near the surface of the nucleus. These results demonstrate that the strains transported across integrins to microfilaments are passed via load-bearing interconnections to microtubules on which the mitochondria anchor, indicating the essential role of mitochondria in mechano-sensing.

The cytoskeleton is the core molecular structure of an active cell and actin microfilaments, microtubules, and intermediate filaments comprise the three major filamentous components of this structure. These filaments link to themselves, to form larger fibrils, and to each other to form structurally coupled networks (Ingber et al., 2014). The fundamental idea of cellular tensegrity is that the cytoskeleton is stabilized by an elastic prestress that is generated and maintained through a complementary force balance between contractile actomyosin filaments and by intracellular structures, such as microtubules, and extracellular binding sites to the extracellular matrix and to other cells (Ingber et al., 1981, 1985; Ingber, 1993, 1997, 2003, 2006). Actin is also the major protein component of the thin filaments in the sarcomere and when myosin, the motor protein binds to actin, substantial mechanical stress is generated on various structures of the sarcomere (Gautel, 2011b). The sarcomeres in striated muscle represent the basic molecular unit for the contractile force. Actin in the thin filaments are crosslinked at the Z-disc, whereas the thick filaments, mainly composed of myosin, are attached to the M-band of the sarcomere that can deliver cross stabilization. The distance between the myosin and actin cross-links, at the M-band and Z-disc, is determined by the giant sarcomeric scaffold titin (aka. connectin). In addition to their mechanical function, these sub-sarcomeric structures and their molecular components are greatly involved in cellular mechanotransduction (Hoshijima, 2006; Linke, 2008). The sarcomeric Z-disc, as well as the M-band, has increasingly been recognized as hubs for signaling pathways that mediate diverse intracellular processes including cell growth and differentiation, as well as protein turnover and gene expression (Hoshijima, 2006; Durieux et al., 2007).

Via the sarcomeric protein telethonin located in the Z-disc, the Z-disc cross talks to growth factors. Further, it has also been shown that telethonin interacts with several other proteins outside the Z-disk and relocalizes in response to stress or atrophy signals (Gautel, 2008). The sarcomeric M-band has also been shown to play a critical role in mechanotransduction and an M-band signaling complex, consisting of titin, Nbr1, p62, and MURF2 mediate gene expression and muscle protein turnover in response to biomechanical stress via the nuclear translocation of the transcription factor serum response factor (SRF) (Lange et al., 2005; Peng et al., 2005; Will et al., 2010). Another member of the M-band proteins, Myosin-interacting M-band-associated stress-responsive protein (myosap, aka. leucine rich repeat containing 39 protein, LRRC39) binds to the myosin tail domain. Will and co-workers demonstrated that myosin is involved in mechanically activated gene transcription via SRF in cardiomyocytes (Will et al., 2010). This indicates that the M-band may contain several mechanostransducers that can control gene expression (Gautel, 2008). Additionally, titin has a kinase domain that is linked to the control of muscle gene expression and protein turnover, located at the M-band of the sarcomere (Lange et al., 2005). Titin was long considered a simple molecular spring, controlling the resting length of sarcomeres. However, it has been established that titin is involved in mechanical signaling pathways in heart muscle (Krüger and Linke, 2009) and several ubiquitin-linked signaling proteins are targeted to, and around the protein kinase domain of titin (Witt et al., 2005; Gautel, 2008).

### Preferential myosin loss in muscle unloading associated with CIM

To date, most studies on the effects of muscle unloading have focused on the effects of removing the external load induced by weight bearing in response to hind limb suspension or microgravity. In immobilized ICU patients, in addition to the removal of the external load by weight bearing, the internal strain in response to activation of contractile proteins is also missing. This results in the complete mechanical silencing, unique for mechanically ventilated, and deeply sedated or pharmacologically paralyzed ICU patients. Microgravity or ground based microgravity models form the basis of research on the effect of muscle unloading-reloading, but the mechanisms and effects may differ from the ICU conditions. The preferential loss of myosin and myosin associated proteins in limb and trunk muscles is a significant characteristic of CIM which separates CIM from other types of acquired muscle weaknesses in ICU patients (Larsson et al., 2000). This phenotype is reproduced in a unique experimental rat ICU model, used in our research group, allowing long-term mechanical ventilation in a deeply sedated, extensively monitored, and pharmacologically paralyzed rats by post synaptic neuromuscular blockade (Norman et al., 2006; Larsson, 2007). This model is not limited by the early mortality (within 1–3 days) observed when commercially available ventilators are used and the longest duration a rat has been continuously monitored and mechanically ventilated to date is 3 months (Dworkin and Dworkin, 1990). In this experimental model, the complete mechanical silencing for long durations, typically longer than 1 week, results in a similar geno- and phenotype as in ICU patients with CIM, i.e., a preferential loss of myosin and myosin associated proteins in parallel with a transcriptional down-regulation of contractile proteins and activation of protein degradation pathways in a typical temporal pattern in limb and trunk muscles (Norman et al., 2006; Ochala et al., 2011a). In accordance with the tensegrity model, mild passive mechanical loading reduces the preferential myosin loss seen in both fast- and slow-twitch muscles as well as the loss of specific force at the single muscle fiber level (Renaud et al., 2013). Similar findings were also observed in response to passive loading in deeply sedated and immobilized ICU patients (Llano-Diez et al., 2012). The significant muscle sparing effect in response to loading is mediated via multiple mechanosensing pathways involving protein degradation pathways and mitochondria resulting in decreased protein degradation, reduced oxidative stress and improved function of mitochondria.

## Protein synthesis in muscle unloading

Removing the pre-stress generated by the complementary force balance, i.e., muscle unloading, induces a rapid decrease in protein synthesis which contributes to the loss of muscle mass (Thomason and Booth, 1990; Han et al., 2007). The primary effect of mechanical stimulation on protein synthesis appears to occur at the level of translational efficiency (Goldspink, 1977; Kimball et al., 2002) and components of the mammalian/mechanistic target of rapamycin (mTOR) play a critical role in the mechanical regulation of protein synthesis and skeletal muscle mass (Thomason and Booth, 1990; Jackman and Kandarian, 2004; Han et al., 2007; Hornberger, 2011). However, different types of mechanical loading/unloading regulates protein synthesis and muscle mass in a distinct manner (Isaacson and Brotto, 2014).

Initially mTOR was identified as a cytoplasmic protein associated with intracellular membranes in early non-muscle studies (Withers et al., 1997; Sabatini et al., 1999) and subsequently shown to associate with the endoplasmic reticulum (ER) and Golgi apparatus in different cell lines (Drenan et al., 2004). Additionally mTOR has been found to be associated with the outer mitochondrial membrane, suggesting a potential role in the regulation of energy metabolism and also demonstrating the involvement of mTOR in the process of mechanotransduction (Desai et al., 2002; Isaacson and Brotto, 2014).

mTOR occurs in two complexes; mTOR complex 1 (mTORC1) and mTOR complex 2 (mTORC2). mTORC1 is the rapamycin sensitive complex that consist of the regulatory associated protein of mTOR (RAPTOR), mammalian lethal with SEC13 protein 8 (mLST8; a.k.a. GbL), the Dep domain-containing mTOR-interacting protein (DEPTOR) and the proline-rich Akt substrate of 40 kDa (PRAS40). In contrast, mTORC2 is the rapamycin-insensitive complex, composed of the rapamycin insensitive companion of mTOR (RICTOR), mLST8, DEPTOR, stress-activated protein kinase-interacting protein 1 (mSIN1) and the protein observed with RICTOR (PROTOR; Weigl, 2012).

Several studies have shown that mTORC1 signaling regulates initiation of translation through the phosphorylation of substrates such as eukaryotic initiation factor (eIF), 4E binding protein-1 (4EBP-1), ribosomal protein S6 (S6), and p70 ribosomal protein S6 kinase (S6K) (Heitman et al., 1991; Haghighat et al., 1995; Hara et al., 1997; Baar and Esser, 1999). These substrates are key regulators of protein synthesis, and are expressed in all cell types (Weigl, 2012). The efficiency of an mRNA is mainly controlled by the eukaryotic initiation factor 4E (eIF4E) which is also part of the eIF4F-complex, together with eIF4G and eIF4A. eIF4A is an RNA helicase which is able to unwind hairpin structures in the 5′untranslated region (UTR) of mRNAs, hence promoting the translational process (Ray et al., 1983; Rogers et al., 2001). 4E-BP1 is an inhibitory phospho-protein, that in the hypo-phosphorylated state, is bound to eIF4E and thereby hindering the association of the eIF4F-complex. When mTORC1 phosphorylates 4EBP-1, 4EBP-1 detaches from eIF4E, increases the formation of the eIF4F complex and improves protein synthesis rate (Weigl, 2012). S6K1 is a positive regulator of protein translation initiation. mTORC1 phosphorylates S6K which in turn phosphorylates several other proteins affecting translation initiation like S6 and eIF4B, another member of the initiation eIF4F-complex (Holz et al., 2005). Thus, the control of translation initiation by mTOR is a key regulator of protein synthesis in skeletal muscle (You et al., 2015).

The upstream regulators of mTORC1 are linked with growth factors, nutrients, energy and stress. Growth factors like insulin and insulin growth factor (IGF) stimulate phosphoinositide 3 kinase (PI3K), which leads to the activation of Akt (aka. protein kinase B, PKB). Activated Akt phosphorylates the tuberous sclerosis complex 2 (TSC2) which is a protein that forms the TSC1/2 complex, together with TSC1 (Inoki et al., 2002). Phosphorylation of TSC2, by Akt, prevent its inhibition of Rheb from promoting mTORC1 activity (Stocker et al., 2003; Weigl, 2012). In addition to Akt, mTORC1 responds to many upstream signals, including amino acids. Further, mTORC1 controls several cellular processes in addition to protein synthesis such as autophagy (Sandri, 2010). Rapamycin inhibits mechanically induced growth and mechanically induced changes in protein synthesis in skeletal muscles (Kubica et al., 2005; Hornberger et al., 2007), this together with studies using transgenic mice models of mTORC1 inactivation indicate that mechanical stimulation of skeletal muscles is sufficient to activate mTORC1, and that the activation of mTORC1 is the basis for the increased protein synthesis and muscle growth observed under mechanical stimulation (Weigl, 2012).

Application of a static stretch to skeletal muscle myotubes *in vitro* significantly increases amino acid transport into stretched cells (Vandenburgh and Kaufman, 1982) which may also activate mTORC1 in an Akt independent manner. Whether the increase in amino acid transport that results from mechanical activation of voltage-sensitive sodium channels contribute significantly to muscle growth or metabolism remains to be tested (Tidball, 2005).

## Muscle degradation in muscle unloading

Activation of proteolytic systems in cells is regulated at the gene level and the transcripts associated with muscle atrophy have been studied by gene expression profiling (Bodine et al., 2001; Gomes et al., 2001). A subset of genes that are commonly up- or down- regulated in muscle atrophy, irrespective of the underlying cause, have been identified and referred to as atrophy-related genes or “atrogenes” (Bodine et al., 2001; Gomes et al., 2001; Lecker et al., 2004; Sacheck et al., 2007). Among these atrogenes there are several transcripts associated with the ubiquitin proteasome system (UPS) and autophagy (Sandri, 2008).

Atrogenes and protein degradation are blocked by the activation of Akt. Akt phosphorylates Forkhead Box (Fox) O, thus inhibiting the UPS. Members of the FoxO family (FoxO1, 3, and 4), downstream Akt, have been identified as key transcription factors controlling the expression of MuRF1 and atrogin-1. Additionally it has been shown that FoxO3, specifically, also regulates autophagy (Sandri et al., 2004; Mammucari et al., 2007; Zhao et al., 2007). Recent work by Milan et al. (2015) demonstrates that muscle-specific deletion of FoxO members protects the muscle from atrophy, further demonstrating the role of FoxOs in the induction of the UPS and the autophagy lysosome pathway. Furthermore, it was demonstrated that FoxOs control several stress-response pathways such as the unfolded protein response, ROS detoxification, DNA repair and translation (Milan et al., 2015).

### Ubiquitin proteasome system

The UPS is an ATP-dependent proteolytic system that targets proteins with ubiquitin (Ub) molecule substrates, through a cascade of conjugating enzymes (ligases), for identification of degradation (Murton et al., 2008). The ubiquitin ligase enzymes (E3) bind the protein substrate and once a protein is ubiquitinated, it is unfolded and fed into the proteasome in an ATP-dependent process (Sandri, 2013). Two important atrogenes that have been found in several animal models of muscle atrophy including unloading are muscle-specific E3s; atrogin-1 and muscle RING finger-1 (MuRF1; Kisselev and Goldberg, 2001). These two atrogenes have been extensively studied in muscle protein degradation and their involvement in muscle atrophy is well known. Mice lacking atrogin-1 and MuRF1 are resistant to muscle atrophy in response to denervation and knockdown of atrogin-1 prevents muscle wasting induced by fasting (Bodine et al., 2001; Cong et al., 2011).

It has been shown that MuRF1 interacts with and controls the half-life of several essential muscle structural proteins like troponin I (Kedar et al., 2004), MyHC (Clarke et al., 2007; Fielitz et al., 2007), and actin (Polge et al., 2011). So far only a few muscle proteins have been identified as substrates for atrogin-1. The substrates found are however all related to growth-associated processes (Tintignac et al., 2005; Csibi et al., 2010).

Further, MuRF1 and atrogin-1 are also upregulated in limb muscles, in response to complete mechanical silencing and the upregulation of these E3 ligases precedes both the muscle atrophy and the preferential myosin loss in response to mechanical silencing (Ochala et al., 2011a; Renaud et al., 2013). In addition, a similar temporal up-regulation pattern of these two atrogenes is also observed in the diaphragm muscle in the experimental rat ICU model, used in our lab, but in the absence of the preferential myosin loss. Even though MuRF1 and atrogin-1 are significant Ub-ligases involved in muscle atrophy, there are likely to be other E3s related to muscle wasting in response to muscle mechanical silencing. Further, the preferential myosin loss in response to the ICU condition cannot be solely explained by the activation of these atrogenes. Particular Ub ligases can be involved in different muscle atrophy models and in different stages of the process. Recently a new set of Ub ligases have been discovered as significant atrogenes that are under the control of the FoxO-family. This set of Ub-ligases includes MUSA1, an E3, that has been found to be critical in muscle atrophy during denervation and fasting (Sartori et al., 2013), Fbxo31, an additional E3, Itch, a Ub-ligase that regulates the half-life of several transcription factors, and Fbxo21, a gene of unknown function but that contains an F-box motif and that has been given the name SMART (Specific of muscle atrophy and regulated by transcription). This novel set of E3s were all up-regulated in response to denervation induced atrophy (Milan et al., 2015).

### Autophagy lysosome pathway

Autophagy is recognized as a crucial protein degradation mechanism in muscle in addition to the ubiquitin–proteasome system (Sandri, 2008), but is also emerging as a novel mechanism in regulating cellular signal transduction by removing activated signaling proteins (Gautel, 2008; Backues and Klionsky, 2011). Autophagy is a physiological process that is vital for the cells to eliminate damaged cell-components and to remodel the cellular architecture (Neel et al., 2013). There are three types of autophagy that all promote proteolytic degradation of cytosolic components; macro-autophagy, micro-autophagy, and chaperone mediated autophagy (CMA). Both macro- and micro-autophagy can ingest large structures through selective and non-selective mechanisms. Macro-autophagy transports cytoplasmic cargo to the lysosome through the intermediate of an autophagosome, a double membrane-bound vesicle. The autophagosome then fuses with the lysosome forming an autolysosome. In contrast, in micro-autophagy, the cytosolic components are taken up by the lysosome directly through invagination of the lysosomal membrane (Glick et al., 2010; Sandri, 2010). In the third type of CMA, proteins that have been targeted are translocated in a complex with chaperone proteins (such as heat shock proteins), and can cross the lysosomal membrane. This way the targeted proteins are recognized by the lysosomal membrane receptor, lysosomal-associated membrane protein 2A (LAMP-2A), and are subsequently unfolded and degraded (Saftig et al., 2008).

Autophagy is primarily considered to be a non-selective degradation pathway, but autophagy can trigger the selective elimination of specific organelles, such as mitochondria (via mitophagy). *Parkin, PINK1, Bnip3, and Bnip3L* are genes that have been identified in mammals to control mitophagy, inactivation of the genes encoding these proteins lead to abnormal mitochondria (Hara et al., 2006). During muscle wasting the mitochondrial network is drastically remodeled in response to fasting or denervation, and autophagy plays an important part in this process (Romanello et al., 2010; Romanello and Sandri, 2010). Alteration in mitochondrial dynamics is sufficient to cause muscle wasting in mice, indicating that disturbance of the mitochondrial network is essential for the muscle homeostasis (Romanello et al., 2010; Romanello and Sandri, 2010).

## Muscle specific differences

Skeletal muscles are heterogeneous at the whole muscle, motor unit and individual muscle fiber levels. The different muscle properties decide the optimal function of each muscle, motor unit and muscle fiber. An important aspect of muscle diversity lies in their embryological origin (Sambasivan et al., 2009; Merrell and Kardon, 2013). Craniofacial muscles, are evolutionarily and developmentally distinct from trunk and limb muscles (Noden and Francis-West, 2006; Sambasivan et al., 2011). Trunk and limb muscles are derived from somites that originate from the paraxial mesoderm of the trunk, whereas the cranial mesoderm gives rise to head muscles like extraocular muscles and cheek muscles like the masseter and the buccinators (Grifone and Kelly, 2007; Nathan et al., 2008; Harel et al., 2009). MyoD, Myf5 and Mrf4 are myogenic regulatory factors that work together to control the entry to the myogenic differentiation program that applies to all skeletal muscles. However, the regulatory hierarchies that act upstream of the myogenic factors are diverse in somatic and cranial mesoderm (Czajkowski et al., 2014). Significant differences between muscles also lies in further development into mature skeletal muscles (Merrell and Kardon, 2013).

In adult skeletal muscles and muscle fibers, the same stimulus can cause divergent responses. The same fiber type may undergo opposite changes in different muscles. Even within the same muscle different fiber types react differently (Blaauw et al., 2013; Schiaffino et al., 2013). The nervous system utilizes the capacity of the muscles to generate force and movement for a variety of motor tasks. These tasks can roughly be divided into three main types; postural joint stabilization, long lasting repetitive activities (like respiration), and fast and powerful actions (such as jumping or kicking). In mammals the motor units are functionally organized into separate components, the motor units each consist of a motoneuron and the muscle fibers that it exclusively innervates. Muscle fibers attain a degree of specialized molecular structure and physiological parameters to perfectly suit the needs of the motor units (Blaauw et al., 2013). The heterogeneity of skeletal muscle fibers, therefore, mainly reflects an adaptation to the different patterns of activity; in addition it also indicates specialization in membrane properties, calcium shuttling mechanisms, contractile machinery, and the structure of the cytoskeleton (Polla et al., 2004; Schiaffino and Reggiani, 2011).

The diaphragm is a muscle engaged in continuous rhythmic activity and diaphragm muscle fibers are characterized by fatigue resistance (Polla et al., 2004; Merrell and Kardon, 2013). In addition to ventilation, the diaphragm muscle is also required for coughing, talking and singing, activities which are phasic and occasional. Diaphragm fibers generally have a smaller CSA than limb muscles. However, the number of capillary vessels surrounding each fiber is the same, the diffusion distance is reduced which makes the oxygen supply more efficient in the diaphragm than in other muscles. This might improve oxygen diffusion and contribute to the increased resistance of the diaphragm to fatigue. Respiratory muscle fibers do not undergo the same changes in response to training and inactivity as limb muscle fibers (Polla et al., 2004; Merrell and Kardon, 2013).

The diaphragm muscle is severely affected in many ICU patients exposed to mechanical ventilation resulting in the rapid development of diaphragmatic weakness due to both atrophy and contractile dysfunction. This harmful effect of prolonged MV has been named ventilator-induced diaphragmatic dysfunction (VIDD; Vassilakopoulos and Petrof, 2004; Powers et al., 2013). Approximately 25% of mechanically ventilated ICU patients experience difficult weaning (Daniel Martin et al., 2013) and the weaning procedures account for 40–60% of the total time on the ventilator (Esteban et al., 1994, 1995). This has significant negative consequences for patient quality of life with an increased risk for pneumonia, mortalaity, and morbidity as well as profound economic consequences for health care providers.

In spite of lack of a preferential myosin loss in the diaphragm, diaphragm muscle fiber function, i.e., fiber atrophy and maximum force normalized to muscle fiber cross-sectional area (specific force), was decreased by more than 85% after 10 days mechanical ventilation in the experimental rat ICU model (Corpeno et al., 2014). An early and maintained oxidative stress in response to controlled mechanical ventilation seems to be a key factor triggering post-translational protein modifications of the myosin molecule, impaired myofibrillar protein organization, and fiber atrophy (Corpeno et al., 2014). The temporal pattern, the phenotype, and mechanism underlying the loss of contractile proteins differ between the diaphragm and limb muscles in response to mechanical ventilation and may in part be related to the passive mechanical loading of the diaphragm by the ventilator, but is most probably also a consequence of other known and unknown factors such as muscle specific differences, oxidative stress, protein modifications and the release of specific factors from the respiratory system (Corpeno et al., 2014).

## Conclusion

The uniquely observed mechanical silencing seen in pharmacologically paralyzed or deeply sedated mechanically ventilated ICU patients is an important factor triggering the specific CIM. Passive mechanical loading significantly alleviates these effects indicating the important role of cellular mechanotransduction in skeletal muscles. There is a growing body of evidence showing that mitochondrial activity plays an important role in the regulation of muscle size and there is emerging evidence demonstrating the important role of mitochondria in mechano signaling in skeletal muscle. The mechanisms controlling the effects of mechanosensation are part of an intricate biological system with important muscle specific differences that needs to be taken into account when designing strategies for reducing muscle wasting and weakness in immobilized and mechanically ventilated ICU patients.

## Author contributions

RCK wrote the manuscript with assistance of LL.

## Funding

This study was financially supported by the Swedish Research Council (8651), the Swedish Foundation for International Cooperation in Research and Higher Education (STINT), the European Commission (MyoAge, EC Fp7 CT-223756 and COST CM1001), King Gustaf V and Queen Victoria's Foundation, and Karolinska Institutet to LL.

### Conflict of interest statement

The authors declare that the research was conducted in the absence of any commercial or financial relationships that could be construed as a potential conflict of interest.
